# Phase Stability of Dross Particles in Hot-Dip Zn-55wt%Al-1.6wt%Si Galvanizing Bath

**DOI:** 10.3390/ma16031211

**Published:** 2023-01-31

**Authors:** Dongdong Qu, Matthew Gear, Qinfen Gu, Nega Setargew, Wayne Renshaw, Stuart McDonald, David StJohn, Kazuhiro Nogita

**Affiliations:** 1 School of Mechanical and Mining Engineering, The University of Queensland, Brisbane 4072, Australia; 2 ARC Research Hub for Australian Steel Manufacturing, Wollongong 2522, Australia; 3 Australian Synchrotron, Australian Nuclear Science and Technology Organisation (ANSTO), Clayton, Melbourne 3168, Australia; 4 Product Innovation and Technology, BlueScope Ltd., Port Kembla, Wollongong 2505, Australia

**Keywords:** hot-dip galvanizing dross, τ5c-Al_20_Fe_5_Si_2_(+Zn), synchrotron X-ray diffraction, phase stability

## Abstract

Dross in a Zn-55wt%Al-1.6wt%Si metal coating bath is a mixture of bath metal and the quaternary intermetallic phase τ5c-Al_20_Fe_5_Si_2_(+Zn). Understanding the properties and formation of dross in a hot-dip Al-Zn galvanizing bath at the processing temperature (~600 °C) is critical for improving the production quality of steel sheet coating. However, dross analysis is usually conducted at room temperature with dross samples taken from the hot-dip bath and it is not known how representative these samples are of the phase(s) existing at high temperature. Using in-situ synchrotron X-ray diffraction (XRD), the crystal lattice and the coefficient of thermal expansion (CTE) of the intermetallic phase have been determined in the temperature range of 30 °C to 660 °C. Phase formation and phase stability of the intermetallic phase in the dross powder have been determined, providing fundamental knowledge for optimizing the production and quality of steel sheet coating.

## 1. Introduction

The Zn-55wt%Al-1.6wt%Si alloy has been used to coat steel sheets for protection against aqueous corrosion, where a multilayered microstructure (steel substrate/intermetallic compound (IMC) layer/coating overlay) is formed [1,2,3,4,5]. Industry has traditionally manufactured this coating by using the continuous hot-dip galvanizing process [1,2]. In the production line, the rolled steel sheet continuously enters the molten Zn-55wt%Al-1.6wt%Si alloy bath (~600 °C), where a metallurgical reaction proceeds between the steel sheet and liquid Zn-55wt%Al-1.6wt%Si alloy, producing an IMC layer [3,4,6]. The IMC layer then provides the heterogeneous nucleation sites for the primary α-Al during the solidification of the coating overlayer after the steel sheet is pulled out of the hot liquid bath [7]. By controlling the jet knives (gas wiping dies to remove excess coating metal) above the hot liquid bath, the required coating overlayer thickness is achieved. It has been shown that as soon as the steel sheet enters the molten Zn-55wt%Al-1.6wt%Si alloy bath, it begins to dissolve, adding Fe to the bath [8]. As the steel sheet continuously passes through the liquid bath, the dissolved Fe accumulates eventually becoming oversaturated. The oversaturated Fe precipitates by forming Al-Fe and Al-Fe-Si containing IMC particles in the liquid bath [8]. It has been reported that these precipitated IMC particles form the dross that either floats as a suspension or settles as sediment in the hot liquid bath [8,9]. It should be noted that apart from the Al-Fe and Al-Fe-Si precipitates, other dross particles are produced by the IMC that forms on top of, but drops off, the steel sheet when it bends around the sink roll in the liquid bath [8,10]. It has been confirmed that the Al-Fe-Si containing IMC formed in the liquid bath and that dropping from the steel sheet have a consistent composition and crystal structure [10].

The liquid Zn-55wt%Al-1.6wt%Si alloy contains Al, Zn, Si and Fe, as shown by the composition. In the liquid state (~600 °C), the strong chemical affinity between Al and Fe produces the binary Al-Fe IMC. Referring to the binary phase diagram, there are several common IMCs frequently observed in the binary Al-Fe alloy, e.g., θ-FeAl_3_ (or Fe_4_Al_13_) and η-Fe_2_Al_5_ [2,4,11,12,13]. With Si involved in the Al-Fe system, additional Al-Fe-Si IMCs were also reported and documented in reference [14]. It was found that in the 55%Al-Zn coating alloy τ5c-Al_20_Fe_5_Si_2_(+Zn) (of cubic crystal unit cell) is the single phase that constitutes the IMC layer as well as the dominant phase component of the dross in the liquid bath [5]. The τ5 IMC in the ternary Al-Fe-Si alloy system is usually denoted as Al_8_Fe_2_Si and has a hexagonal close-packed (hcp) lattice structure [15,16,17]. However, the τ5 IMC has been found to transform from the hcp lattice to the body-centered cubic (bcc) lattice as τ5c-Al_20_Fe_5_Si_2_(+Zn) where a large amount of Zn is absorbed in the τ5c [3,4,6,18,19,20]. While the hcp τ5 crystal structure for Al_8_Fe_2_Si is well determined [15,16,17], the crystal structure for τ5c is little reported, particularly with a certain proportion of Zn atom substituting for the other atoms in the large crystal lattice [19,20].

In the hot-dipped low carbon steel sheet coated by the Zn-55wt%Al-1.6wt%Si alloy, the τ5c IMC layer forms at the liquid bath temperature and maintains a stable phase down to room temperature [6,21,22]. The performance of this coating product proves the stability of the τ5c IMC which bonds the coating overlayer to the steel substrate. However, the Zn containing τ5c in the dross particle is little studied, with little work carried out to evaluate the stability of this τ5c IMC at the liquid bath temperature, ~600 °C. Therefore, in this paper, the dross powder collected from the liquid Zn-55wt%Al-1.6wt%Si alloy bath in hot-dip galvanizing is studied using in-situ synchrotron X-ray diffraction (XRD) at a variety of temperatures. The phase stability of the dross powder during heating up to 660 °C is revealed.

## 2. Materials and Methods

Dross particles were collected from the bottom of a liquid Zn-55wt%Al-1.6wt%Si alloy bath in the hot-dip galvanizing production line at BlueScope Steel Ltd. (Western Port, Victoria, Australia). The method to prepare the dross particles is described elsewhere [8]. After further grinding, the dross particles were placed in a quartz capillary of 0.7 mm in diameter. The capillary tube was then mounted onto a sample stage for synchrotron XRD measurements. To protect the powder sample from oxidation during heating, the sealed capillary tube was pumped to a high vacuum (1 × 10^−4^ mbar) before switching on the XRD recording system. The high vacuum condition was maintained during the entire synchrotron XRD measurement.

In-situ synchrotron XRD measurement was conducted at the Powder Diffraction beamline of the Australian Synchrotron. A monochromatic X-ray with a photon energy of 21 keV, which gives a wavelength of 0.5888 Å, was used as the incident beam. The whole measurement was set in a Debye–Sheerer transmission powder diffraction mode. The diffraction signals were recorded by a Mythen microstrip detector moving between two positions within 5°, by setting the exposure time to be 5 min at each measuring temperature. The optical system for the synchrotron XRD was calibrated by measuring a standard LaB_6_ sample (NIST660b, *a* = 4.15689 Å, Pm$\overset{–}{3}$m, particle size 2–40 μm) at room temperature. The sample was step-linearly heated from 30 °C to 660 °C, at a heating rate of 20 °C/min. The temperature during the measurement was controlled by using a hot blower. The diffraction patterns were recorded at a 30 °C interval during heating. Afterwards, the diffraction patterns were analyzed by using the PDViPeR v2.0.1 software and the TOPAS-Academic V6 package [23]. The fundamental parameters of the crystal lattice structure were collected in the TOPAS-Academic V6 package based on the Pawley fitting. The Pawley fitting was carried out to determine the lattice size of τ5c based on a bcc unit cell. Synchrotron radiation wavelength calibration (0.59 Å), 2θ zero error and instrument configuration functions were determined based on the standard LaB_6_ powders. The Pseudo Voight function was used to define the peak shape of XRD patterns for the Pawley fitting. The background of the synchrotron XRD pattern was fitted by using the Chebyshev function with 6 coefficients as used in the TOPAS-Academic V6 package. Phase identification of the α-Al, β-Zn and FeAl_3_ was carried out with the assistance of the PDF-4+ 2018 database of the International Centre for Diffraction Data (ICDD). The PDF cards for α-Al, β-Zn and FeAl_3_ are PDF 00-004-0787, PDF 00-004-0831 and PDF 00-047-1420, respectively.

## 3. Results and Discussion

### 3.1. Phase Transformation of the Dross Particles

**Figure 1:**
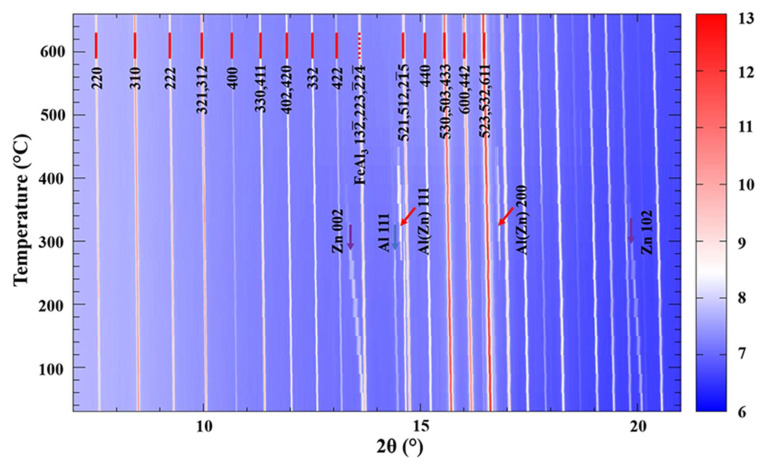
Two-dimensional temperature sequential maps of synchrotron XRD for the dross powder during heating. The logarithmic intensity for each diffraction pattern can be referred to the color scale to the right of the map. Several typical lattice plane reflections are indexed and labelled in this map for the bcc lattice of τ5c, as well as the mixed α-Al, β-Zn and FeAl_3_ powder.

Figure 1 shows the two-dimensional temperature sequential map of the synchrotron XRD patterns for the dross particles during the heating. For the in-situ synchrotron XRD analysis, the phase transformation can be determined by tracking the diffraction peak positions and intensity variation [24,25,26]. In Figure 1, the variation of the diffraction peaks is reflected by the white strip lines where the dominant synchrotron XRD patterns are shown to evolve steadily during heating from 30 °C to 660 °C. This indicates that the primary IMC in those dross particles remains in a stable crystal lattice, even at the hot liquid bath temperature (~600 °C). The thermal expansion of the IMC is also reflected by the shift of the diffraction peak position, particularly at the high 2θ range (18–21°). In addition to the dominant XRD for the IMC, a small amount of Al and Zn was involved in the phase transformation, as revealed by the variation of the indexed diffraction peaks for α-Al and β-Zn.

The identification of the XRD pattern was carried out with the assistance of the TOPAS-Academic V6 package. The typical lattice plane reflections at low angles are indicated in Figure 1. It is found that the dross particles contain the τ5c, FeAl_3_, α-Al and β-Zn phases. It has been reported that τ5c has a bcc unit cell in the manufactured coating product [19]. The synchrotron XRD in the current study confirms that τ5c is also the dominant IMC in the dross particles formed in the hot liquid bath in the production line. It should be noted that the τ5c has a large solubility of Zn and other elements [3,4,5,6,18,19,20], which can be stable in the liquid bath and down to room temperature. The identification of the τ5c bcc lattice demonstrates that there are two bcc lattices of different sizes for the τ5c in the currently studied dross particles. This is confirmed by the following XRD pattern fitting by using the Pawley method. As such, the τ5c is indexed by using two bcc crystal lattices with *a*_1_ = 12.5743(3) Å and *a*_2_ = 12.5631(8) Å at 30 °C, respectively, in space group Im-3.

Besides the τ5c, FeAl_3_ is also found in the dross particles. The FeAl_3_ is determined using the monoclinic crystal lattice with *a* = 15.4875(5) Å, *b* = 8.0850(7) Å and *c* = 12.4921(2) Å at 30 °C, in space group C2/m. As indicated by the diffraction peak at 2θ = 12.7°, the strongest diffraction peak for FeAl_3_ was recorded for the overlapped (13$\overset{–}{2}$), (223) and (22$\overset{–}{4}$) lattice plane reflections. The FeAl_3_ is another IMC that forms in the current Al-Zn-Si-Fe alloy system at high temperatures due to the strong chemical affinity between Fe and Al [27]. The small amount of FeAl_3_ IMC is most likely those FeAl_3_ particles that form in the liquid Zn-55wt%Al-1.6wt%Si alloy bath and remain in the dross particles before the completion of the phase transformation from FeAl_3_ to τ5c.

The dross particles were taken from the liquid Zn-55wt%Al-1.6wt%Si bath and washed by using HF acid to select the IMC particles from the main Al-Zn alloy. A small amount of α-Al and β-Zn particles were trapped inside the dross powders during the procedure. As shown in Figure 1, the α-Al and β-Zn phases are also indexed and shown by the indicated arrows. The α-Al is indexed using a face-centered cubic (fcc) crystal lattice with *a* = 4.0482(8) Å at 30 °C, in space group Fm-3m. The β-Zn is indexed using a hexagonal close-packed (hcp) crystal lattice with *a* = 2.6647(2) Å and *c* = 4.9489(9) Å at 30 °C, in space group P63/mmc. The (002) and (102) lattice plane reflections for β-Zn and the (111) and (200) lattice plane reflections for α-Al in this dross powder are clearly recorded in the XRD patterns from 30 °C to 450 °C during the phase transformation.

**Figure 2:**
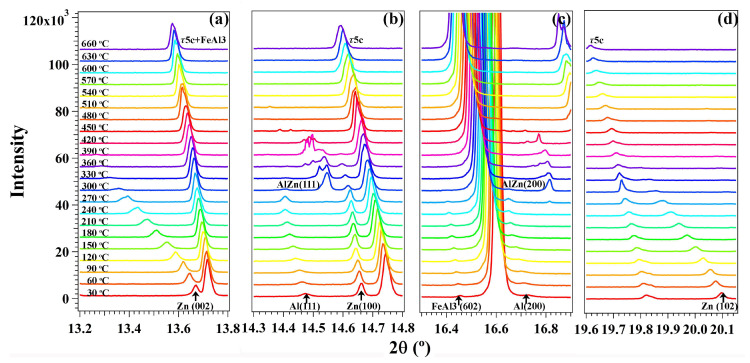
The XRD patterns to highlight the lattice plane reflections for (**a**) β-Zn(002), (**b**) α-Al(111), (**c**) α-Al(200) and (**d**) β-Zn(102). The strongest diffraction peaks are indexed to be the overlapped reflections of τ5c(431)+(510)+(501) and FeAl_3_(13$\overset{–}{2}$)+(223)+(22$\overset{–}{4}$) in (**a**); τ5c(521)+(512) in (**b**); τ5c(523)+(532)+(611) in (**c**) and τ5c(721)+(712)+(552)+(633) in (**d**). The XRD patterns at different temperatures are offset for the clarity.

Figure 2 shows the details of the phase transformation for α-Al and β-Zn in the dross powder. The selective plots for the β-Zn(002), β-Zn(102), α-Al(111) and α-Al(200) demonstrate that the low-temperature α-Al transformed into high-temperature Al(Zn) during heating, α-Al+β-Zn→Al(Zn). This agrees with the eutectoid reaction in the pure binary Al-Zn alloy system, where this reaction occurred at 277 °C [28]. Nonetheless, it is shown that this reaction begins at 270 °C in the dross powders. Considering the 30 °C temperature interval for the current experiment, this reaction could begin at a temperature between 240 °C and 270 °C, which can be confirmed in future work. By tracking the intensity of the diffraction peaks for α-Al, β-Zn and Al(Zn), it is found that the β-Zn phase remained from 30 °C to 270 °C and decreased in amount from 270 °C to 390 °C. The β-Zn phase completely disappeared above 390 °C. The α-Al remained from 30 °C to 270 °C and decreased from 270 °C to 330 °C. Above 330 °C, the low-temperature α-Al completely transformed into the high-temperature Al(Zn). The Al(Zn) started to form at 270 °C. Then, Al(Zn) increased until the complete consumption of α-Al and remained together with β-Zn from 330 °C to 390 °C. After that, only Al(Zn) remained until 450 °C and disappeared above 450 °C. Therefore, it is shown by in-situ synchrotron XRD that the reaction of α-Al+β-Zn→Al(Zn) occurs in the temperature range of 270–330 °C. This is caused by the multi-component phase equilibrium in the current Al-Zn-Si alloy system. From 330 °C to 390 °C, the phase transformation is for the Al(Zn) to absorb the remaining β-Zn in the dross powders. Above 450 °C, the disappearance of Al(Zn) may be caused by the dissolution of Al(Zn) into the τ5c IMC.

### 3.2. Variation of the Crystal Lattice Parameters during Heating

#### 3.2.1. Whole XRD Pattern Fitting by the Pawley Method

**Figure 3:**
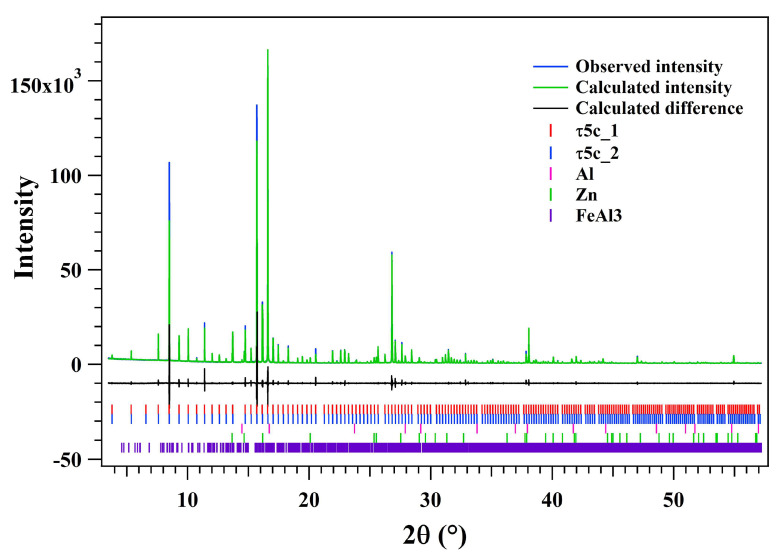
Typical one-dimensional XRD pattern for the dross powder at 30 °C. The observed intensity is presented in blue. The calculated intensity after Pawley refinement is shown in green. The fitting difference (black curve) and the phase index (vertical line markers) are shown in the figure as well. The fitting difference curve is offset for clarity.

As shown above, there are four types of crystal lattices with five groups of lattice parameters indexed in Figure 1. To reveal the lattice parameter changes during the heating of the dross powders, a Pawley fitting was performed on the in-situ synchrotron XRD patterns from 30 °C to 660 °C. The 2θ range selected for the fitting is from 3.5° to 57.5°. The crystal lattice structure information (i.e., space group and lattice parameters) for the fitted crystals is shown above for τ5c, FeAl_3_, α-Al and β-Zn at 30 °C. Figure 3 shows the typical 1-dimensional synchrotron XRD patterns obtained after the Pawley fitting at 30 °C. A *R*_wp_ of 8.03% was obtained for this fitting with α-Al, β-Zn, FeAl_3_ and τ5c. The two types of bcc lattice for τ5c index markers can be seen as similar to each other but with a small displacement due to the slightly different lattice parameters. Following the same fitting procedure, the whole batch of the in-situ synchrotron XRD patterns for the dross powders was fitted using the Pawley method. All the fitted lattice parameters are shown in the Supplementary Table S1. The *R*_wp_s for all the fittings are between 4.94–9.39% which provides a high level of trust for evaluating the change in the lattice parameter.

#### 3.2.2. The Lattice of τ5c

**Figure 4:**
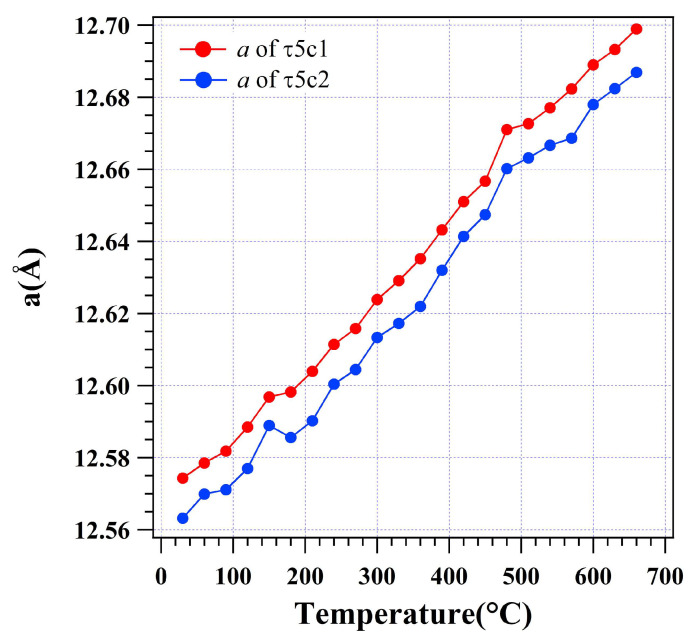
Lattice parameters for the τ5c bcc unit cell based on the size *a* in the dross powder.

Figure 4 shows the variation of the lattice parameters for the τ5c bcc crystal lattice during heating. The two fitted bcc lattices, τ5c1 and τ5c2, both expand in the heating process. It is noted that there is no interaction between τ5c1 and τ5c2. The unit size difference for the τ5c1 and τ5c2 is nearly constant as demonstrated by the nearly constant gap between the two plots versus temperature. It is noted that both τ5c1 and τ5c2 showed an abrupt increase of the lattice at 150 °C and a slight decrease above 150 °C. This indicates that some solute absorption and rejection occurred in the big lattices of τ5c1 and τ5c2. The abrupt increase of *a* for τ5c1 and τ5c2 at 480 °C (*R*_wp_ = 6.17%) indicates another phase transformation. In Figure 2, it is confirmed that the Al(Zn) disappears above 450 °C. The abruptly increased lattice parameter of τ5c1 and τ5c2 is the evidence to demonstrate that τ5c absorbed Al and Zn into the large bcc lattice at high temperature. It is found that the size *a* of τ5c1 increases from 12.5743(3) Å to 12.6989(5) Å as temperature increases from 30 to 660 °C, with *a* = 12.6889(6) Å at 600 °C. The size *a* of τ5c2 increases from 12.5631(8) Å to 12.6869(1) Å as temperature increases from 30 to 660 °C, with *a* = 12.6779(4) Å at 600 °C. The linear thermal expansions of τ5c1 and τ5c2 are then calculated to be 0.99% and 0.98% from 30 to 660 °C, respectively. Considering the solute absorption, the linear coefficients of thermal expansion (CTEs) for τ5c1 and τ5c2 are both fitted to be α_[100]_ = 1.65 × 10^−5^ K^−1^.

#### 3.2.3. The Lattice of FeAl_3_

**Figure 5:**
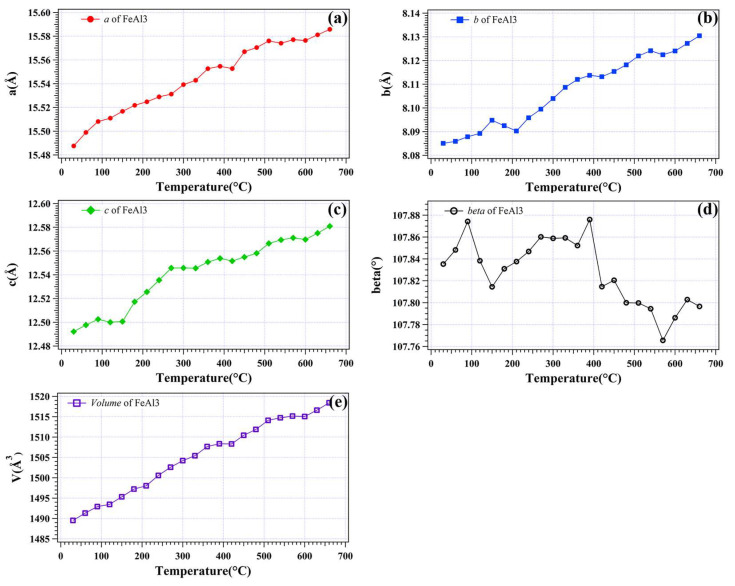
Lattice parameters for the FeAl_3_ monoclinic lattice based on the size (**a**) *a*, (**b**) *b*, (**c**) *c*, (**d**) *beta* and (**e**) unit cell volume in the dross powder.

The variation of the lattice parameters *a*, *b*, *c* and *beta* for the monoclinic crystal lattice of FeAl_3_ in the heating process is shown in Figure 5. The *a*, *b*, *c* and *beta* of the monoclinic lattice are fitted to be 15.4875(5) Å, 8.0850(7) Å, 12.4921(2) Å and 107.8353°, respectively, at 30 °C for the FeAl_3_ in the dross powder. The a, *b* and *c* increased to 15.5857(8) Å, 8.1304(5) Å and 12.5809(1) Å, respectively, at 660 °C. The *beta* of the FeAl_3_ crystal lattice is noted to fluctuate and decrease slightly from 107.83(5)° at 30 °C to 107.79(6)° at 660 °C. This indicates that the monoclinic crystal lattice for FeAl_3_ in this dross powder relaxed during the thermal expansion with temperature. As shown in Figure 4, an abnormal lattice change is observed for τ5c, α-Al and β-Zn at 150 °C. A similar change in the lattice of FeAl_3_ is also observed at this temperature but with different behavior. While *b* increases abruptly at 150 °C, *c* is noted to decrease. There is no apparent change of *a* except a linear thermal expansion. However, *beta* shows a decrease at this temperature as well. Considering the unit cell volume (Figure 5e), the lattice changes for FeAl_3_ at 150 °C might be caused by the atom redistribution inside the lattice but not solute rejection. At 420 °C, the *a*, *b*, *c* and *V* for FeAl_3_ lattice all decreased with thermal expansion, while *beta* is shown to decrease. This reveals some solute substitution in the FeAl_3_ lattice that is caused by the phase transformation. The linear CTEs for the *a*, *b* and *c* of FeAl_3_ monoclinic lattice are fitted to be α_[100]_ = 1.50 × 10^−4^ K^−1^, α_[010]_ = 7.61 × 10^−5^ K^−1^ and α_[001]_ = 1.41 × 10^−4^ K^−1^, respectively. *Beta* is fitted to decrease at a rate of−1.05 × 10^−4^°/K. The synchrotron XRD in Figure 1 and Figure 3 shows that FeAl_3_ contribute a limited fraction of the dross powder where only the strongest lattice plane reflections are recorded. Unlike the α-Al, β-Zn and Al(Zn), the FeAl_3_ is shown to be stable in the dross powder from 30 to 660 °C with only a slight change in the lattice size caused by the solute absorption/rejection. Due to the complicated phase transformation in the FeAl_3_, future in-depth work is required to clarify the changes of the crystal structure of FeAl_3_ in future.

#### 3.2.4. The Lattice of α-Al

**Figure 6:**
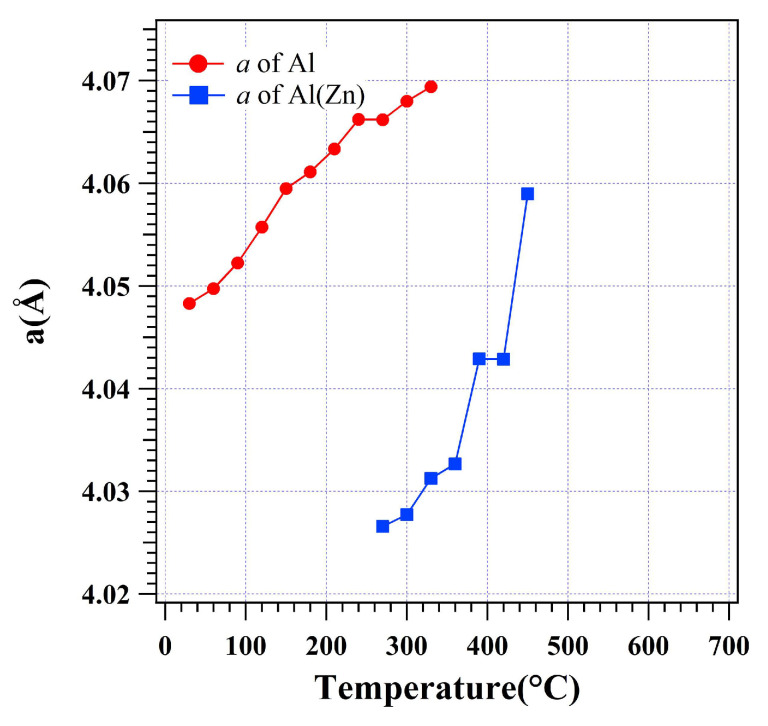
Lattice parameters for the low-temperature α-Al and high-temperature Al(Zn) fcc unit cells based on size *a* in the dross powder.

Figure 6 shows the variation of the lattice parameter *a* for the fcc crystal lattices of low-temperature α-Al and high-temperature Al(Zn) during heating. The *a* of the fcc lattice expands from 4.0482(8) Å at 30 °C to 4.0661(9) Å at 270 °C. When the Al(Zn) forms, the *a* of Al(Zn) is fitted to be 4.0265(7) Å at 270 °C. The lattice size *a* of Al(Zn) shrinks by 0.97% compared with the *a* = 4.0661(9) Å of α-Al at 270 °C. For α-Al, the lattice is shown to linearly expand from 30 to 270 °C. The linear CTE for α-Al is fitted to be α_[100]_ = 7.43 × 10^−5^ K^−1^. The slight increase of *a* at 150 °C indicates that α-Al also absorbed a small amount of solute similar to τ5c and β-Zn in the dross powder. Above 390 °C, only Al(Zn) remained in the dross powder during heating. The CTE for Al(Zn) increases after α-Al is completely transformed into Al(Zn) at 330 °C from α_[100]_ = 7.27 × 10^−5^ K^−1^ to α_[100]_ = 2.63 × 10^−4^ K^−1^. While the CTE of Al(Zn) below 330 °C is nearly the same as that of α-Al, it is around 3 times larger above 330 °C. The low-temperature α-Al is shown to contain a certain amount of Zn referring to the binary phase diagram, while the high-temperature Al(Zn) is shown to contain a large amount of Zn [28]. It is the substitution of Zn for the Al in the Al(Zn) fcc crystal lattice that causes the shrinkage of lattice for Al(Zn) at 270 °C. After the low-temperature α-Al is completely transformed into Al(Zn), it is noted that Al(Zn) stays together with the remaining β-Zn from 330 to 390 °C (Figure 1 and Figure 2). Therefore, the increased CTE for Al(Zn) is most likely caused by the remaining β-Zn diffusing into the Al(Zn) lattice. It is noted that two levelling-offs appear in α-Al and Al(Zn) at 270 °C and 420 °C, respectively, shown in Figure 6. This change of the lattice size reflects that the thermal expansion of the lattice is balanced with the solute absorption occurring in both α-Al and Al(Zn). At 270 °C, the decrease of *a* in α-Al occurs due to the phase transformation of α-Al+β-Zn→Al(Zn). The small size Zn atoms most likely diffuse into the α-Al lattice and this caused the slight shrinkage of the lattice size of α-Al. At 420 °C, the shrinkage of Al(Zn) happens immediately after the remaining β-Zn is completely consumed at 390 °C. Considering the change of the lattice of FeAl_3_, this shrinkage of Al(Zn) is most likely caused by the Al redistribution from Al(Zn) to FeAl_3_.

#### 3.2.5. The Lattice of β-Zn

**Figure 7:**
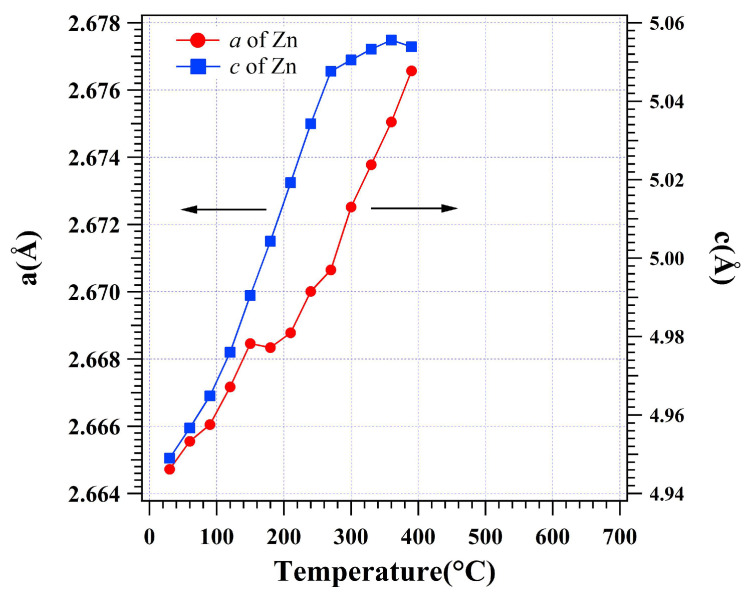
Lattice parameters for the β-Zn hcp unit cell based on sizes *a* and *c* in the dross powder.

Figure 7 shows the variation of the lattice parameters *a* and *c* for the β-Zn hcp crystal lattice during heating. The *a* increases from 2.6647(2) Å at 30 °C to 2.6765(7) Å at 390 °C. At 150 °C, *a* has an increased change similar to the expansion of τ5c1 and τ5c2. This indicates that the hcp lattice of β-Zn absorbed some solute along the *a* axis in the basal plane. However, during the Al(Zn) formation, *a* keeps a constant rate of linear expansion. It can be fitted that the linear CTE for *a* of β-Zn is α_[100]_ = 3.14 × 10^−5^ K^−1^. The *c* increases from 4.9489(9) Å at 30 °C to 5.0538(7) Å at 270 °C almost linearly before the formation of Al(Zn). The linear CTE of *c* for β-Zn from 30 to 270 °C can be fitted to be α_[001]_ = 4.25 × 10^−4^ K^−1^. As heating from 270 to 360 °C, the increase rate of *c* slows down to α_[001]_ = 8.88 × 10^−5^ K^−1^. Above 390 °C, the β-Zn is completely absorbed into the Al(Zn). It can be seen that the lattice expansion of β-Zn increases much faster in the *c* axis than in *a*. Despite the lattice expansion of the β-Zn in heating, it should be noted that the amount of the β-Zn phase in the dross powder decreased during the formation of Al(Zn) above 270 °C. After the formation of Al(Zn), the lattice expansion of β-Zn becomes nearly five times slower in the *c* axis, while the *a* axis keeps expanding at the same rate. As known in the α-Al+β-Zn→Al(Zn) reaction, β-Zn transformed into the solute in the Al(Zn) lattice. The slower expansion of β-Zn might be caused by the solute depletion from the β-Zn lattice before β-Zn is completely consumed.

As shown above, α-Al and β-Zn underwent a phase transformation in the heating of the dross powder, while the dominant τ5c and the tiny amount of FeAl_3_ IMCs remain a stable lattice structure from room temperature up to 660 °C. This indicates that τ5c IMC has good phase stability at the liquid bath temperature (~600 °C) during hot-dip galvanizing. It should be noted that the current in-situ synchrotron XRD is performed on the isolated particles which are enclosed in the quartz capillary tube. The environment of those tested dross particles is different from those in the molten alloy bath where the τ5c particles are most likely in equilibrium with the liquid Zn-55wt%Al-1.6wt%Si alloy at 600 °C. The presence of transition metal trace elements in an industrial molten alloy bath may change the τ5c particles in some way, as inferred by the segregation of Cr and V to the IMC layer on the steel strip [5]. Therefore, the chemical homogeneity is critical to controlling the dross formation in the liquid bath during hot-dip galvanizing.

## 4. Conclusions

The dross powder from the bottom of a liquid Zn-55wt%Al-1.6wt%Si alloy bath in hot-dip galvanizing was investigated using in-situ synchrotron powder XRD under vacuum conditions at a variety of temperatures. The dross powder was indexed to primarily contain the τ5c-Al_20_Fe_5_Si_2_(+Zn). The bcc lattice of the τ5c was found to be stable from 30 to 660 °C. The α-Al, β-Zn and FeAl_3_ were also found in the dross powders. Due to the high solubility of Al and Zn in τ5c, the bcc lattice of two different sizes was used to index the τ5c IMC. The τ5c and FeAl_3_ IMCs were stable in heating from 30 to 660 °C, while α-Al and β-Zn underwent the α-Al+β-Zn→Al(Zn) reaction at 270–330 °C. The high-temperature Al(Zn) solid solution dissolved into τ5c above 450 °C. The bcc lattice sizes for the two τ5cs are fitted to be *a*_1_ = 12.5743(3) Å and *a*_2_ = 12.5631(8) Å, respectively, at 30 °C. The linear CTE for those two τ5cs is fitted to be α_[100]_ = 1.65 × 10^−5^ K^−1^. This work has provided considerable insight into the physical and chemical properties of the IMC phase in the dross, which improves the process control and management of dross build-up in the hot-dip liquid metal pot. Since the τ5c particles have higher mass density than the melt, dross particles present in the melt will agglomerate to form larger particles. Once they reach a critical size, they will settle to the bottom of the pot and form a layer of bottom dross. To remove bottom dross, a costly and specialized industrial practice referred to as Zn-enrichment is employed. In the Zn-enrichment process, zinc ingots are added to the coating bath to increase the density of the melt. The consolidated mass of bottom dross is then broken up by jack-hammering, and the broken pieces of bottom dross float to the surface due to the increased density differences between the Zn-enriched melt and the broken-up pieces of dross. The removal process is a huge safety concern and a high-cost practice. The work we are reporting has led to an improved understanding of the nature of the intermetallic phase and its stability at the process temperature of 600 °C and will significantly improve the management of bottom dross build-up in the coating pot.

## Data Availability

The XRD raw data presented in this study are available on request from the corresponding author. The analyzed data presented in this study are available in Supplementary Materials.

## Supplementary Materials

The following supporting information can be downloaded at: https://www.mdpi.com/article/10.3390/ma16031211/s1, Table S1: The fitting results of the lattice parameters for T5c, Al, Zn and FeAl_3_ in the dross powder by Pawley method.
