# Supplementary material for: Phase Stability of Dross Particles in Hot-Dip Zn-55wt%Al-1.6wt%Si Galvanizing Bath
Source: Materials (Basel). 2023 Jan 31;16(3):1211. doi: 10.3390/ma16031211 (PMC9921612; doi:10.3390/ma16031211)
Supplement: Supplementary file 1 [file materials-16-01211-s001.zip › materials-2157299-SI.pdf]

Table S1. The fitting results of the lattice parameters for T5c, Al, Zn and $FeAl_3$ in the dross powder by Pawley method.

Temperature(°C)	τ5c_1	τ5c_2	Zn		$FeAl_3$				Al	Al(Zn)	R_{wp}(%)
	Im-3	Im-3	$P6_3$/mmc		C2/m				Fm-3m	Fm-3m	
	a(Å)	a(Å)	a(Å)	c(Å)	a(Å)	b(Å)	c(Å)	beta(°)	a(Å)	a(Å)	
30	12.57433	12.56318	2.66472	4.94899	15.48755	8.08507	12.49212	107.8353	4.04828	0	8.02733
60	12.57855	12.5699	2.66555	4.95667	15.49892	8.0859	12.49771	107.8482	4.04973	0	8.08803
90	12.58181	12.57109	2.66605	4.96486	15.50811	8.08783	12.50254	107.8743	4.05223	0	8.50604
120	12.58848	12.57697	2.66717	4.976	15.51099	8.08926	12.50003	107.8383	4.05572	0	8.66935
150	12.59686	12.58892	2.66846	4.99049	15.51676	8.09479	12.50061	107.8145	4.05947	0	9.39366
180	12.59824	12.58561	2.66834	5.00429	15.52179	8.09249	12.51728	107.831	4.06111	0	8.0053
210	12.60394	12.59021	2.66878	5.01923	15.52481	8.09025	12.52556	107.8375	4.06333	0	7.97991
240	12.61143	12.60041	2.67001	5.03426	15.52892	8.09584	12.53544	107.8468	4.06622	0	7.63421
270	12.61584	12.60446	2.67065	5.04763	15.53123	8.09949	12.54569	107.8602	4.06619	4.02657	7.45793
300	12.62388	12.61333	2.67252	5.05052	15.5392	8.10394	12.54572	107.8588	4.06798	4.02771	7.45506
330	12.62911	12.61724	2.67378	5.05327	15.54287	8.10869	12.54552	107.8592	4.06939	4.03124	8.23736
360	12.63518	12.62194	2.67505	5.05559	15.55264	8.11203	12.55074	107.8522	0	4.03266	8.02431
390	12.6432	12.632	2.67657	5.05387	15.55467	8.11374	12.55382	107.876	0	4.04289	6.3004
420	12.65101	12.6414			15.55271	8.11318	12.55161	107.8147	0	4.04285	6.56411
450	12.65673	12.64744			15.56695	8.11537	12.55491	107.8205	0	4.05897	5.67985
480	12.67104	12.66018			15.57047	8.11819	12.55813	107.7999	0	0	6.17028
510	12.67268	12.66316			15.57608	8.12193	12.5664	107.7997	0	0	5.84162
540	12.67708	12.66668			15.57407	8.12413	12.56937	107.7944	0	0	5.18826
570	12.68231	12.66864			15.57709	8.12243	12.5711	107.7656	0	0	5.91427
600	12.68896	12.67794			15.57645	8.12402	12.56967	107.7862	0	0	4.93966
630	12.69325	12.68238			15.58115	8.1272	12.57509	107.8028	0	0	5.86342
660	12.69895	12.68691			15.58578	8.13045	12.5809	107.7965	0	0	6.82692
